# A New View of the Last Universal Common Ancestor

**DOI:** 10.1007/s00239-024-10193-w

**Published:** 2024-08-09

**Authors:** Aaron D. Goldman, Arturo Becerra

**Affiliations:** 1 Department of Biology, Oberlin College, Oberlin, OH USA; 2 Blue Marble Space Institute of Science, Seattle, WA USA; 3 Facultad de Ciencias, UNAM, Mexico City, Mexico

**Keywords:** Last universal common ancestor, Time tree analysis, Universal paralogs, Tree reconciliation, Ancient metabolism, Ancient ecology

The last universal common ancestor (LUCA) is one of the earliest stages of evolution that can be studied using standard phylogenetic methods. Recent decades have seen numerous studies aiming to date LUCA's emergence (Betts et al. 2018), reconstruct its genome and proteome (Becerra et al. 2007; Goldman et al. 2013), and predict its metabolism (Weiss et al. 2016; Goldman et al. 2023; Ledford and Meredith 2024), habitat (Weiss et al. 2016), and ecological context (Krupovic et al. 2020). While preparing the special issue on the LUCA for the Journal of Molecular Evolution, a new and innovative study by Moody et al. (2024) was published. We describe this study and situate it within the broader context of early evolutionary history.

The study by Moody et al. presents several analyses on the timing, proteome, genome, environment, and ecology of the LUCA. Dating the LUCA or any ancient ancestor is a complex and uncertain research problem. Molecular clocks based on constant mutation rates are unreliable due to variations over time and across lineages. Proteins used to date the LUCA must also be under strong negative selection, further obscuring the relationship between the number of mutations and geological time. A recent method calibrates molecular clocks using horizontal gene transfers that can be identified on phylogenetic trees and that correspond to a geochemical signature or fossil that can be dated (Wolfe and Fournier 2018). Moody et al. take advantage of similar fossil-based time constraints (Betts et al. 2018) while also calibrating their molecular clock using universal paralog protein families (Shih and Matzke 2013).

Universal paralogs are protein families that duplicated before the last universal common ancestor (LUCA), resulting in paralogous proteins in the LUCA proteome (Kollman and Doolittle 2000; Zhaxybayeva et al. 2005). In a phylogenetic tree of a universal paralog family, each paralog forms a separate clade with its own LUCA node. By comparing the time estimate of the LUCA node across both paralog sub-trees of the same family, the time estimate can be cross-calibrated. Moody et al. applied calibrated molecular clock analysis to a collection of five well-established universal paralog trees and estimate the date of the LUCA to be ~ 4.2 Ga (4.09–4.32 Ga). This dating is interesting because it leaves very little time for the LUCA to emerge. The moon-forming impact likely occurred around 4.5 Ga (Fu et al. 2023), and the first habitable environments likely formed around 4.3 or 4.4 Ga (Wilde et al. 2001; Miyazaki and Korenaga 2022). Therefore, the 4.2 Ga dating, if correct, suggests that early evolution, from the origin of life to the LUCA, may have occurred within only 100–200 million years.

This finding is particularly surprising given that the LUCA also appears to represent an evolutionary stage in which organisms were already as complex as some modern prokaryotes (Becerra et al. 2007). Numerous studies have attempted to reconstruct the genome or proteome of the LUCA, and most depict it as possessing a DNA genome, a complete translation system, a cell membrane, and a complex metabolism. However, despite this general consensus, the specific predictions of individual studies often diverge, rendering them somewhat unreliable (Crapitto et al. 2022). This inconsistency likely results from a lack of best practices in LUCA proteome reconstruction studies. Specifically, no previous LUCA proteome study has employed both taxonomically broad sampling and paired gene tree-species tree comparisons, which are essential for detecting horizontal gene transfers.

In this context, the Moody et al. study represents a significantly more rigorous approach to LUCA proteome reconstructions. The study used tree reconciliation analysis on 9365 protein families, comparing each protein tree to a species tree to estimate gene duplications, gene losses, and horizontal gene transfers (Szöllõsi et al. 2013). This allowed the authors to assign probabilities of being present in the LUCA proteome for each protein family. Using the strictest criteria, 399 protein families were identified as present in the LUCA proteome, which is similar in number to other recent reconstructions, such as 355 protein families from Weiss et al. (2016) and 366 protein families from Crapitto et al. (2022). However, Moody et al. also used the probabilities for all 9365 protein families to estimate that the LUCA proteome likely comprised 2451–2855 proteins. This estimate, though much higher than previous reconstructions, aligns with the idea that LUCA had a complexity similar to modern, free-living prokaryotes, which typically encode thousands of proteins (Sela et al. 2016).

This probable LUCA proteome allowed the authors to describe the LUCA's metabolism, physiology, habitat, and ecology. The metabolic reconstruction includes pathways common across the tree of life, such as glycolysis/gluconeogenesis, the citric acid cycle, and nucleotide biosynthesis. It also features a nearly complete Wood–Ljungdahl Pathway, used by modern archaea and bacteria for acetogenic growth and carbon fixation. The presence of this pathway could indicate an organoheterotrophic acetogenic metabolism, further suggesting that the LUCA would necessarily have been part of an ecosystem with autotrophic organisms that provided it with organic compounds. If, on the other hand, the LUCA had a chemoautotrophic acetogenic metabolism, the authors suggest that its most likely habitats would have been either hydrothermal vents or the ocean surface. However, key enzymes in early CO_2_ fixation pathways and the overlap between methanogenic, acetogenic, and autotrophic pathways, such as the CO dehydrogenase/acetyl-CoA synthase, complicate a definitive conclusion about LUCA's metabolism (Becerra et al. 2014). Despite this, the study by Moody et al., with its comprehensive phylogenetic representation of basal lineages in both prokaryotic domains, is a significant step toward uncovering the metabolic capabilities of the LUCA and determining whether it relied on acetogenic growth or carbon fixation.

The reconstructed LUCA proteome includes physiological features such as DNA synthesis, aminoacyl-tRNA biosynthesis, ribosome formation, a cell membrane, and membrane-based ATP synthesis. One of the more surprising results of the reconstructed LUCA physiology is the presence of a CRISPR-Cas system, indicating that an immune system against viral infection had already evolved by the time of the LUCA. Therefore, Moody et al. depict the LUCA as a complex cellular organism with a sophisticated genetic system, advanced energy metabolism, and viral defense mechanisms. This LUCA likely lived in one of two potential ocean environments, coexisting with other organisms whose descendants either did not survive or have yet to be discovered. This depiction of a physiologically and ecologically complex LUCA provides strong evidence that this stage of evolutionary history was far removed from the origin of life.

This new study of the LUCA marks a significant advance in our understanding. However, although we have a probable list of protein families present in the LUCA, further research is required to determine the specific molecular functions of their ancient ancestors. Understanding these functions would not only support predictions about the LUCA’s habitat and ecology but also deepen our understanding of early evolutionary processes. Additionally, a simplified characterization of the LUCA, focusing on traits under strong natural selection, is generally more robust and testable than a detailed catalog of metabolic genes. Viewing the LUCA as a population of cells, and the reconstructed LUCA proteome as representing a core genome of a broader pangenome (Goldman and Kaçar 2023), can lead to valuable insights into early evolution and diversification mechanisms (Estrada et al. 2022). To this end, the disparity described by Moody et al. between the probable LUCA proteome (399 protein families) and the estimated number of encoded proteins in the LUCA genome (2451–2855 proteins) offers an enticing lead toward a broader, more nuanced understanding of the LUCA.

In sum, Moody et al. provide a detailed and reliable depiction of the LUCA. But in doing so, their study raises important new questions that will no doubt be the focus of early evolution research in the near future.
